# Audiovisual associations alter the perception of low-level visual motion

**DOI:** 10.3389/fnint.2015.00026

**Published:** 2015-03-31

**Authors:** Hulusi Kafaligonul, Can Oluk

**Affiliations:** ^1^National Magnetic Resonance Research Center (UMRAM), Bilkent UniversityAnkara, Turkey; ^2^Department of Psychology, Bilkent UniversityAnkara, Turkey

**Keywords:** motion perception, visual motion processing, audiovisual associations, multisensory, direction discrimination

## Abstract

Motion perception is a pervasive nature of vision and is affected by both immediate pattern of sensory inputs and prior experiences acquired through associations. Recently, several studies reported that an association can be established quickly between directions of visual motion and static sounds of distinct frequencies. After the association is formed, sounds are able to change the perceived direction of visual motion. To determine whether such rapidly acquired audiovisual associations and their subsequent influences on visual motion perception are dependent on the involvement of higher-order attentive tracking mechanisms, we designed psychophysical experiments using regular and reverse-phi random dot motions isolating low-level pre-attentive motion processing. Our results show that an association between the directions of low-level visual motion and static sounds can be formed and this audiovisual association alters the subsequent perception of low-level visual motion. These findings support the view that audiovisual associations are not restricted to high-level attention based motion system and early-level visual motion processing has some potential role.

## Introduction

Perception is shaped by both immediate pattern of sensory inputs and our prior experiences with the external world. Phenomena such as adaptation and learning have been used extensively to understand how prior experiences over different timescales are involved in shaping visual motion perception. Of particular interest is that associative learning (the process by which an association between two stimuli is learned) revealed unexpected levels of sensory plasticity and changes in visual motion processing (Schlack and Albright, 2007; Schlack et al., 2007). However, these studies mostly focused on a single sensory modality (i.e., vision). As learning in natural settings typically involves multisensory input, such unisensory learning might not be optimal for engaging learning mechanisms adapted to operate in multisensory environments. Consistent with this ecological argument, multisensory associations have, in a series of recent studies, been discovered to have dramatic influences on the perception of visual motion (for a review see Shams et al., 2011).

A recent paradigm developed by Teramoto et al. (2010) provides compelling evidence that multisensory associations influence visual motion perception^1^. In their study, two circles were flashed side by side with a temporal offset at the right of a fixation target. The flashed circles induced a perception of apparent motion (i.e., a circle moving either from left-to-right or right-to-left) when the spatial and temporal offset between them were small. Expectedly, a static flickering circle was perceived if the circles were presented with a temporal offset at the same spatial location. During a typical association phase, observers viewed these apparent motion displays alternating between rightward and leftward directions. Each flashed circle of apparent motion was synchronized with a brief static sound of distinct frequency. The static sounds led to two different sound sequences and each one of these sequences was paired with a motion direction. After the association phase, the sounds biased the perceived direction of apparent motion and even induced motion perception of a static flickering circle in the direction as previously paired with the given sound sequence during the association phase. Accordingly, brief static sounds determined the perceived visual motion direction and this driving effect acquired through associations lasted for at least a few days. Using a similar paradigm, Hidaka et al. (2011) showed that, after an audiovisual association phase, static sounds are also able to change the perceived direction of random dot displays. As opposed to previous associative learning studies (e.g., Haijiang et al., 2006), these studies provide a clear demonstration that associations between static sounds and visual motion direction can be established in a short amount of time and they do not require any conditional reinforcement. Moreover, these audiovisual associations have been found to be selective for certain stimulus features (e.g., visual field, sound frequency), suggesting that they occur at the perceptual level rather than any decision level. However, the neural mechanisms and processing stages underyling them remain unclear within the well studied visual motion hierarchy. An obvious question to ask is whether such associations are restricted to later stages of visual motion processing and only mediated by higher-order sensory areas.

The existence of two basic motion systems is suggested by many studies^2^. First one, low-level pre-attentive motion energy system, is dependent on the spatiotemporal (positional) changes in luminance (Anstis, 1980; Braddick, 1980; Adelson and Bergen, 1985). The low-level motion energy is believed to be computed by the motion detectors located at lower motion areas. Moreover, a number of studies have shown that attentive tracking of salient visual features can overcome ambiguity in low-level spatio-temporal cues and generate consistent visual motion percept (Lu and Sperling, 1995a; Culham et al., 2000). It has been proposed that attentive tracking constitutes a high-level attention based motion system mediated by higher-order motion areas (Cavanagh, 1992; Lu and Sperling, 1995b). As mentioned above, in order to create motion, previous audiovisual association studies on visual motion perception used either a single visual object or random dots not very well controlled for tracking of displaced features. Such stimuli can be localizable in space and activate both low-level motion energy and high-level attention-dependent positional tracking mechanisms. In this respect, they do not provide any specific information about the neural mechanisms underlying audiovisual associations.

In the current study, we aimed to determine whether audiovisual associations could be formed and affect visual motion perception in absence of higher-order attentive tracking mechanisms. If so, this outcome would imply that audiovisual associations are not restricted to later stages of visual motion processing (i.e., high-level attention based motion system). We took two basic approaches to minimize the involvement of higher-order attentive tracking and to isolate low-level pre-attentive visual motion processing. The involvement of high-level processes can be changed by manupilating the parameters of random-dot displays (Sato, 1998). For instance, when the dot size is decreased and the dot density is increased, it becomes difficult to attentively track the position of individual dots and hence, high-level processes become less effective (Ho and Giaschi, 2009a,b). Therefore, we first optimized the parameters of our random dot displays to minimize any explicit attention-dependent position tracking of individual dots. In addition, using reverse-phi illusion has also been found to be a fruitful approach to restrict the involvement of higher order processes (Azzopardi and Hock, 2011; Kafaligonul and Stoner, 2012; Hock and Nichols, 2013). In this illusion, perceived direction of motion is opposite to the physical displacement and consistent with the outcome of low-level motion energy mechanisms rather than high-level attentive tracking of displaced features (Anstis, 1970). Accordingly, we also used reverse-phi random dot motions in our psychophysical experiments.

## Materials and Methods

### Participants

Ten observers (four females, six males; age range: 21–32 years) participated in this study with eight being naive to the purpose of the experiments. There were five observers in Experiment 1 and six observers in Experiment 2. Only one of the naïve observers took part in both experiments. All participants had normal hearing and normal or corrected-to-normal visual acuity. Participants gave informed consent, and all procedures were in accordance with international standards (Declaration of Helsinki) and approved by the ethics committe at Faculty of Medicine, Ankara University.

### Apparatus and Stimuli

We used Matlab version 7.12 (Mathworks) with Psychtoolbox 3.0 (Brainard, 1997; Pelli, 1997) for stimulus presentation and data acquisition. The precision of visual and auditory stimuli was achieved by high-performance video (NVIDIA GeForce Graphics) and sound (Asus Xonar ASIO Compatible) cards. Visual stimuli were presented on a 21″ LCD monitor (NEC MultiSync 2190UXp, 1600 × 1200 pixel resolution and 60 Hz refresh rate) at a viewing distance of 57 cm. A SpectroCAL photometer was used for luminance calibration and gamma correction of the display. Sounds were emitted by headphones (Sennheiser HD 518) and amplitudes were measured by a sound-level meter (SL-4010 Lutron). Head movements were constrained by a chin rest. All experiments were performed in a dark room.

A small red circle (12 arc-min diameter) at the center of the display served as a fixation target. Visual motion stimuli were rotating dynamic random dot displays and consisted of ten random-dot frames presented within an annulus aperture (inner radius: 2.25^∘^, outer radius: 4.5^∘^). The dot density of each random-dot frame was 33 dots/deg and each dot had a 3.15 arc-min diameter. For regular phi motion, all dots were either lighter (51.53 cd/m^2^) or darker (0.62 cd/m^2^) than the gray background (26.19 cd/m^2^). We used the same luminance values for reverse-phi motion. However, instead of a having fixed dot luminance throughout the duration of motion, the contrast polarity of dots were reversed in each of the two consecutive random-dot frames. The duration of each random-dot frame was 100 ms and there was no temporal interval between each frame for both motion types. To control the strength of motion signal and determine the position of every dot in each random-dot frame, we used white noise motion algorithm orginally developed by Britten et al. (1992). In brief, the motion signal was generated by randomly selecting a percentage of the dots (“coherent dots”) from each random-dot frame to be replotted at a shifted location in a single direction [clockwise (CW) or counter-clockwise (CCW)]. The remaining dots (“noise dots”) were replotted at random positions in the next random-dot frame. Even though this manipulation resulted in noise dots with random speed and direction, the coherent dots moved in the single direction with a constant speed (15^∘^ rotation per second). Based on the algorithm, the lifetime of coherent motion dots was probabilistic outcome of motion coherence (Pilly and Seitz, 2009; Schutz et al., 2010). When the proportion of coherent dots were low, it became hard to distinguish them from dynamic noise dots due to their short lifetimes. However, the lifetimes of coherent dots got longer as their proportion was increased. For the 100% coherent dots condition, all dots moved in a single direction and stayed on the display during the presentation of motion. Auditory stimulus was a static tone of either low (500 Hz) or high (2000 Hz) frequency and it was presented for 1 s (same as visual motion duration) at 83 dB SPL. Each tone burst was windowed with a 10 ms rise-and-fall time and sampled at 44.1 kHz.

### Procedure

**FIGURE 1:**
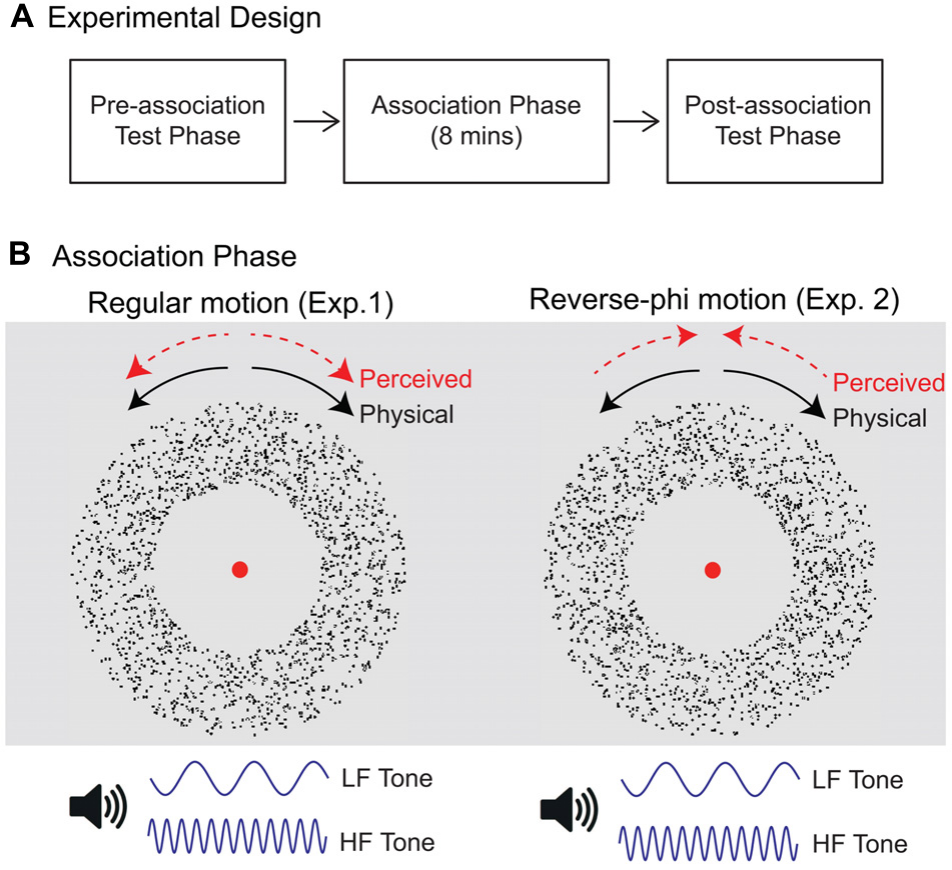
**(A)** Experimental design. Each experiment consisted of three consecutive stages: pre-association test phase, association phase and post-association test phase. Pre-association (Post-association) test phase: assessment of pre-association (post-association) direction discrimination for rotating stimuli either presented with static sounds or alone. Association phase: pairing of rotation directions with static sounds of distinct frequencies. **(B)** Stimuli used during the association phase. In Experiment 1, regular motion was presented with static tones. Each rotation direction [clockwise (CW) or counter-clockwise (CCW)] was paired with either low-frequency (LF) or high-frequency (HF) tones. Reverse-phi motion was used in Experiment 2. The perceived direction of rotation (as shown by dotted red arrows) was in the direction opposite to the physical displacement (black arrows).

The procedure was similar to the one reported in Hidaka et al. (2011). Each experiment consisted of three separate stages: pre-association test phase, association phase, post-association test phase (**Figure 1A**). During each association phase, the directions of motion stimuli with 100% coherent dots were paired with static tones of distinct frequencies (**Figure 1B**). In Experiment 1, regular random dot motion (either lighter or darker than the background) were presented with static tones. Three observers viewed CW and CCW rotating stimuli with low (CW sound condition) and high (CCW sound condition) frequency tones, respectively. The pairing of motion directions and static tones was reversed for the remaining observers. Each direction of motion was pseudorandomly presented 120 times. Therefore, there were 240 total presentations and each association phase (including the time interval between each presentation) lasted for around 8 min. New random dot displays were generated for each presentation. Observers were asked to attend to both rotating stimuli and static tones while they were fixating on the red circle at the center of the display.

The pre- and post-association test phase were exactly the same and each of them included two separate experimental sessions: regular and reverse-phi motion session. In each experimental session, visual motion stimuli (either regular phi or reverse-phi) rotated in either CW or CCW direction with coherence level chosen pseudorandomly from six values: 5, 15, 25, 35, 50, and 80%. Moreover, each experimental session had a balanced mixture of two auditory stimulus conditions: CW (static tone paired with CW direction during the association phase) and CCW (static tone paired with CCW direction during the association phase) sound conditions, and one visual-only (no tone) stimulus condition. Every stimulus was presented eight times per session. Accordingly, there were 288 trials (3 audiovisual conditions × 6 motion coherence levels × 2 motion directions × 8 trials per stimulus) in each experimental session. Observers engaged in a motion direction discrimination task (two-alternative forced-choice). They fixated on the red circle at the center of the display. They were instructed neither to attend to dots (individual or groups) nor to track their displacement. They were told that some visual motion stimuli would be accompanied by tones and asked to pay attention to both motion and tones. New random dot displays were generated for each trial to rule out tracking of previously identified dot patterns. At the end of each trial, observers indicated, by pressing left or right arrow keys, whether the moving stimuli rotated CW or CCW direction. Each observer completed four experimental sessions (2 test phases × 2 motion types) and the order of phi and reverse-phi sessions were randomized for each testing stage. Prior to these experimental sessions, each participant was shown examples of visual-only regular and reverse-phi stimuli followed by a practice session for each motion type.

We used the same procedure and stimuli in Experiment 2 except for the motion stimuli used during the association phase. In the association phase of Experiment 2, reverse-phi motion stimuli with 100% coherent dots were used. The perceived direction (opposite to the physical displacement) of reverse-phi motion and static tones of distinct frequencies were paired (**Figure 1B**). Therefore, two sound conditions were defined based on the perceived direction of the reverse-phi motion.

### Data Analysis

For auditory and visual-only conditions, a cumulative Gaussian function was fitted to the single and group-averaged data by using psignifit (version 2.5.6), a software package that implements the maximum likelihood method described by Wichmann and Hill (2001a,b). The 50% point on the resultant curves yields the point of subjective equality (PSE). PSE is the motion coherence level for which CW and CCW motion directions are equiprobable. We used the standard deviation (SD) of the fitted cumulative Gaussian as a measure of the slope of psychometric function and direction discrimination sensitivity. As the SD decreases, the psychometric curves become steeper indicating a better direction discrimination and a lower direction discrimination threshold. To quantify the effect of each static sound on PSE values, we calculated the PSE shifts by subtracting the PSE values for the visual-only condition from the ones for each sound condition. Moreover, in order to assess the sound specific changes in direction discrimination, we computed slope changes by subtracting the SD values for the visual-only condition from the ones for each sound condition. We applied a repeated measures ANOVA and follow-up tests for simple main effects on these difference values computed from individual observers.

## Results

### Experiment 1: Regular Motion Direction and Static Tone Association

Observers were not able to discriminate the direction of regular random dot motion when the proportion of coherent dots was low. As the proportion of coherent dots (i.e., motion coherence level) was increased, they perceived the direction of motion in the direction of physical displacement, and the direction discrimination performance of each observer was improved (**Figures A,B**). Motion coherence level also had a similar effect on reverse-phi direction discrimination. However, unlike in regular motion, observers perceived the direction of reverse-phi motion in the direction opposite of the displaced dots, thus inconsistent with the tracking of dot positions (**Figures C,D**). The perceived direction of reverse-phi motion is instead consistent with the outcome of low-level motion energy mechanisms (Adelson and Bergen, 1985), which have been considered to be implemented within early levels of motion processing (Emerson et al., 1987; Krekelberg and Albright, 2005). Even though it is very difficult to track the position of random dots in our regular motion stimuli, the perceived direction in reverse-phi motion illusion provided us with a more explicit approach to check whether audiovisual associations affect the activity of low-level motion mechanisms.

**FIGURE 2:**
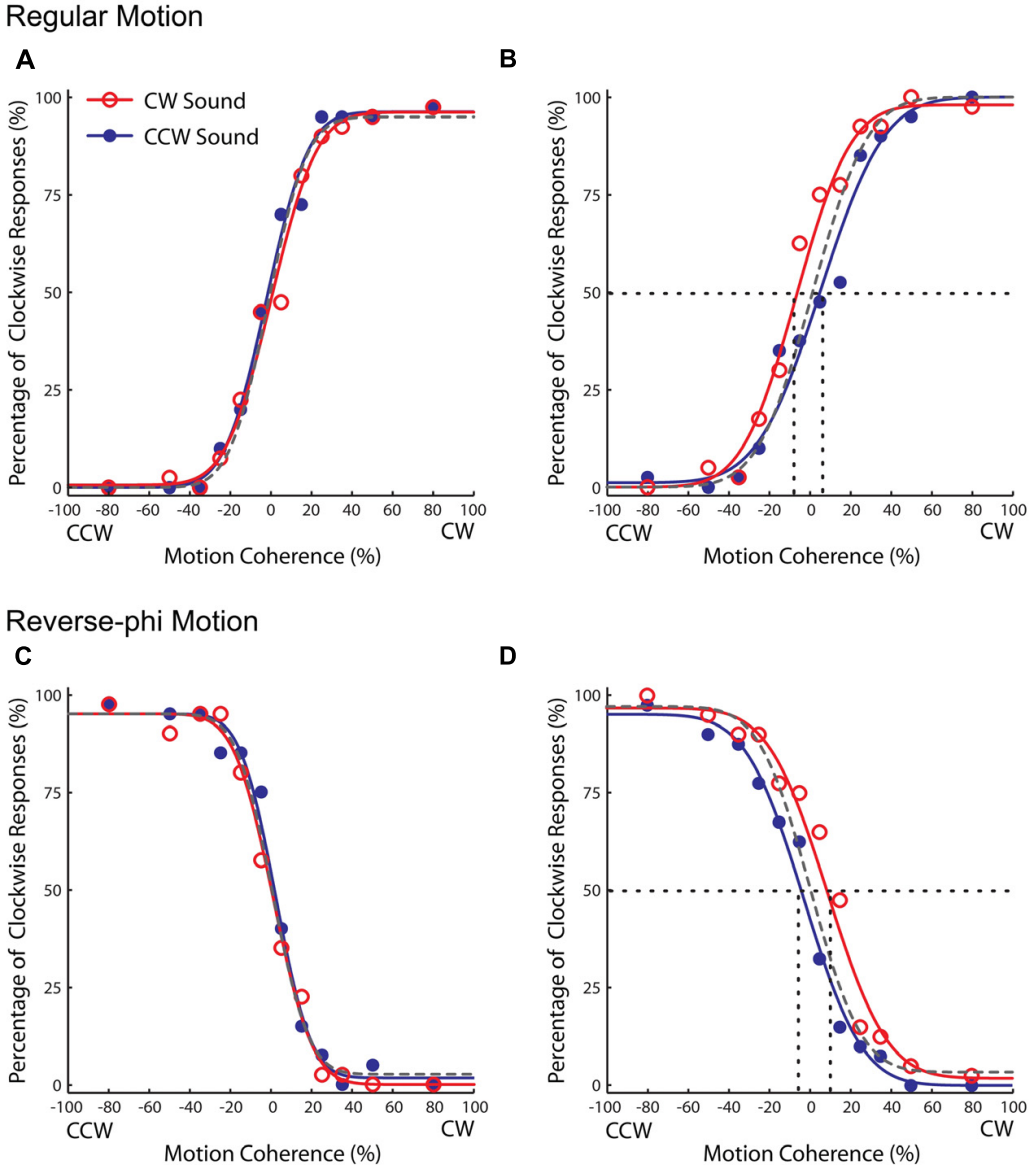
**Results of Experiment 1 (regular motion used in the association phase).** Group averaged data (*n* = 5, four were naïve observers) for different experimental conditions. Top panel: pre-association **(A)** and post-association **(B)** data for regular motion. Bottom panel: pre-association **(C)** and post-association **(D)** data for reverse-phi motion. Each plot indicates the proportion of trials in which the visual stimulus was judged to rotate in clockwise direction. The negative and positive values on the horizontal axis correspond to CCW and CW directions, respectively. The open and filled symbols represent different sound conditions. To avoid clutter, the data for the visual-only condition were not shown and the dashed gray curve corresponds to psychometric fit for that condition. The intersection of the 50% point with the vertical line gave an estimate of the PSE for each condition.

For both motion types, static tones did not cause any change in the psychometric curves at the pre-association phase. After the paired presentation of motion directions and static tones during the association phase, the static tones significantly biased the perceived direction of regular motion in favor of the exposed audiovisual pairing. In the post-association phase, the static tone paired with the CW direction (CW sound condition) led to an increase in the CW direction reports relative to the one paired with the CCW direction (CCW sound condition). Therefore, the psychometric curve for the CW sound condition was shifted to negative coherency values and its PSE value was smaller than the CCW sound condition (**Figure 2B)**). More importantly, each static tone also baised the perceived direction (opposite to the physical displacement) of reverse-phi motion in the associated direction. As in the regular motion, the CW sound condition increased the CW direction reports of the reverse-phi motion direction and the CW direction reports decreased for the CCW sound condition. This led to an increase in the PSE value of the psychometric curve relative to the one for the CCW sound condition (**Figure 2D)**).

**FIGURE 3:**
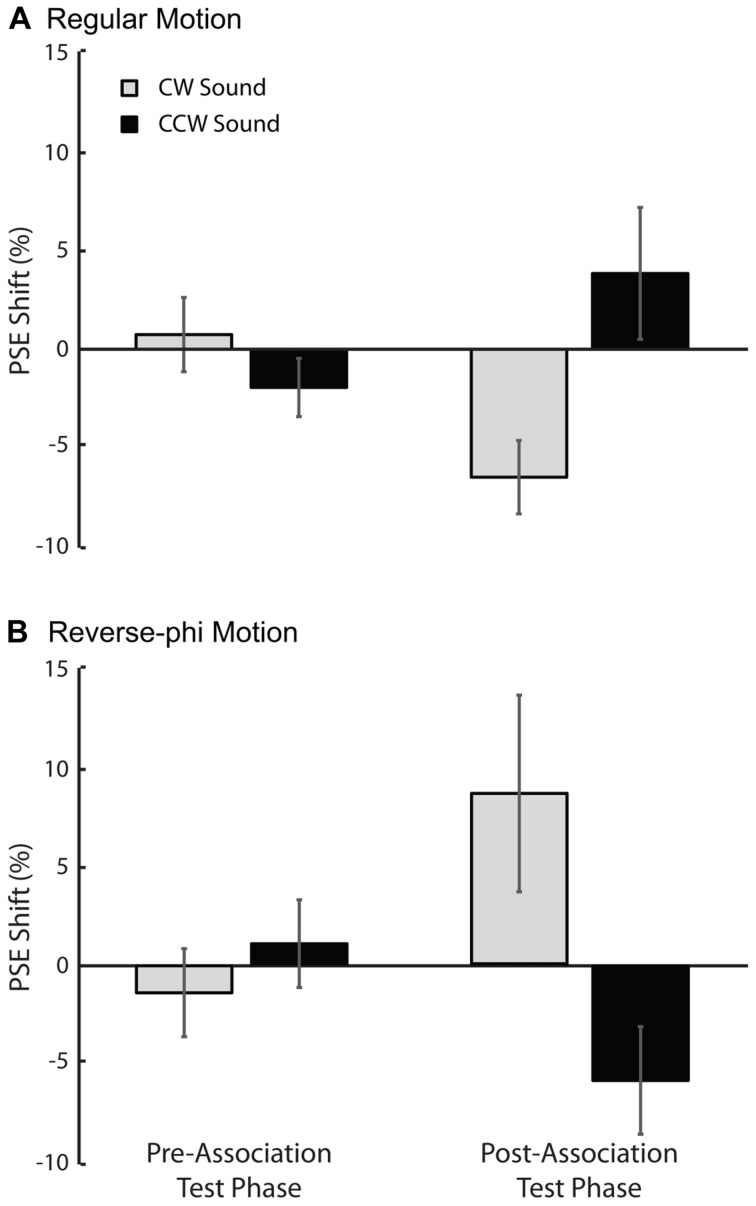
**Results of Experiment 1 (regular motion used in the association phase).** The averaged PSE shifts (in units of motion coherence level) of all observers as a function of test phase. **(A)** Pre-association and post-association data for regular motion. **(B)** Pre-association and post-association data for reverse-phi motion. The gray and black bars represent CW and CCW sound conditions, respectively. Error bars indicate +SEM.

In support of the aformentioned observations above, a statistical analysis of the shifts in PSE values for each sound condition relative to the visual-only showed only a significant three-way interaction [three-way repeated-measures ANOVA motion type, test-phase and sound condition as factors, *F*(1,4) = 11.132, *p* < 0.05]. The main effects of motion type [*F*(1,4) = 0.008, *p* = 0.934], test phase [*F*(1,4) = 0.547, *p* = 0.501] and sound condition [*F*(1,4) = 1.107, *p* = 0.352] were not significant. We tested for simple main effects to understand the exact nature of the three-way interaction. For regular phi motion, the follow-up tests revealed a significant effect of sound on PSE shifts in the post-association test [**Figure 3A**, *F*(1,4) = 19.655, *p* < 0.05] but not a significant effect in the pre-association test phase [*F*(1,4) = 0.834, *p* = 0.413]. In the post-association test of reverse-phi motion, the PSE shift for CW sound condition was also significantly greater than the one for CCW sound in the expected direction [**Figure 3B**, *F*(1,4) = 6.371, *p* < 0.05]. There was no significant difference between sound conditions in the pre-association test phase [*F*(1,4) = 2.362, *p* = 0.199]. In addition, we also estimated PSE shifts for bright and dark conditions of regular motion separately. Our tests revealed non-significant effects of dot polarity and its interactions with the other factors (Supplementary Table S1).

### Experiment 2: Reverse-phi Motion Direction and Static Tone Association

We used 100% coherent rotating dots for the association phase of Experiment 1. Basically, the dots stayed on the display during the presentation of each motion presentation. Due to this long lifetime of dots, observers could have conceivably attended to random dot patterns in the 100% rotating regular motion and then noted positional changes of these patterns. Therefore, it is possible that the rotating stimuli may have activated attention based position tracking. To determine whether any position tracking is necessary for motion direction and static tone association in a more controlled way, we used reverse-phi motion in the association phase of Experiment 2.

**FIGURE 4:**
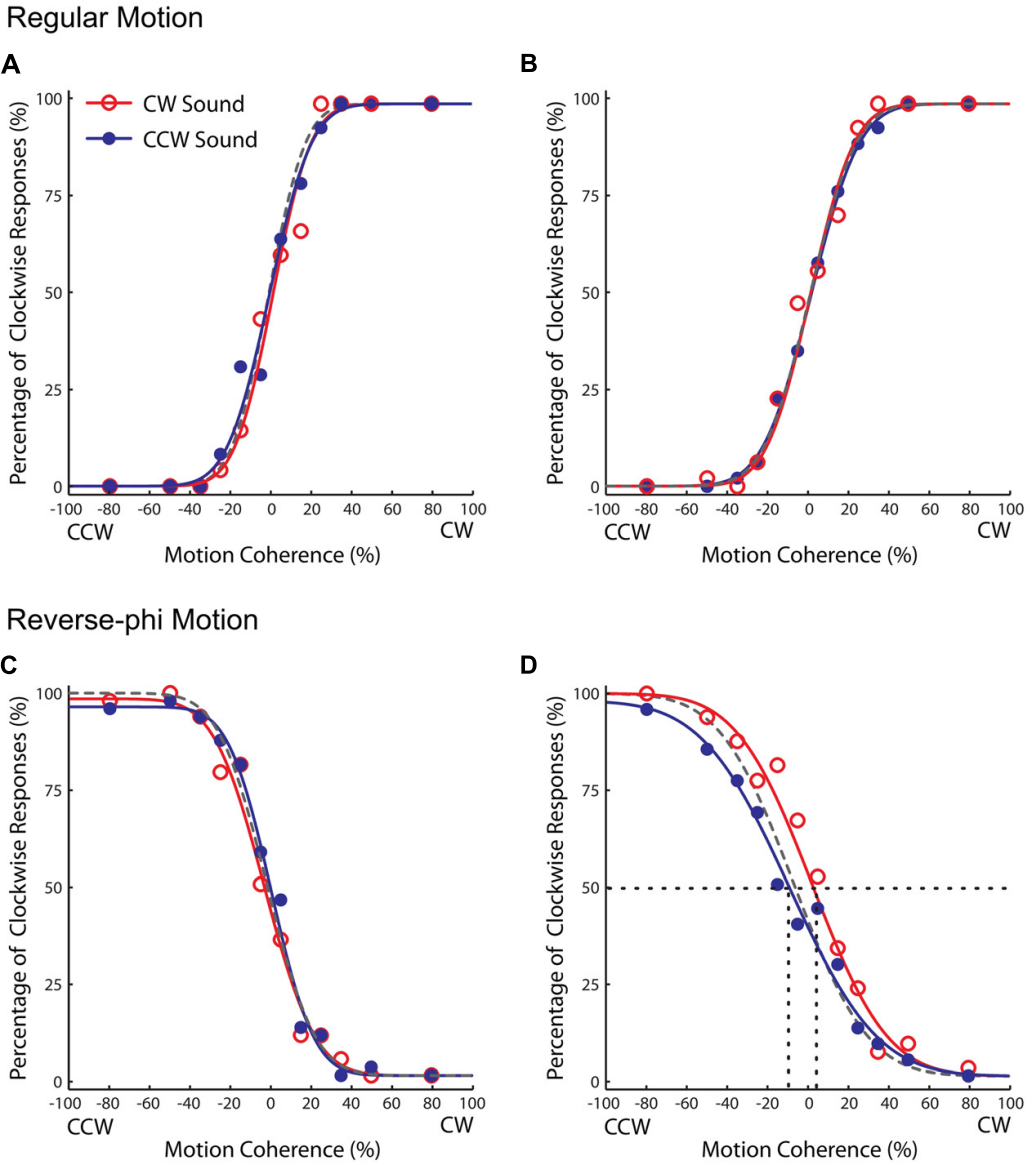
**Results of Experiment 2 (reverse-phi motion used in the association phase).** Group averaged data (*n* = 6, five were naïve observers) for different experimental conditions. Top panel: pre-association **(A)** and post-association **(B)** data for regular motion. Bottom panel: pre-association **(C)** and post-association **(D)** data for reverse-phi motion. Other conventions are the same as those in **Figure 2)**. Each plot indicates the proportion of trials in which the visual stimulus was judged to rotate in clockwise direction.

**FIGURE 5:**
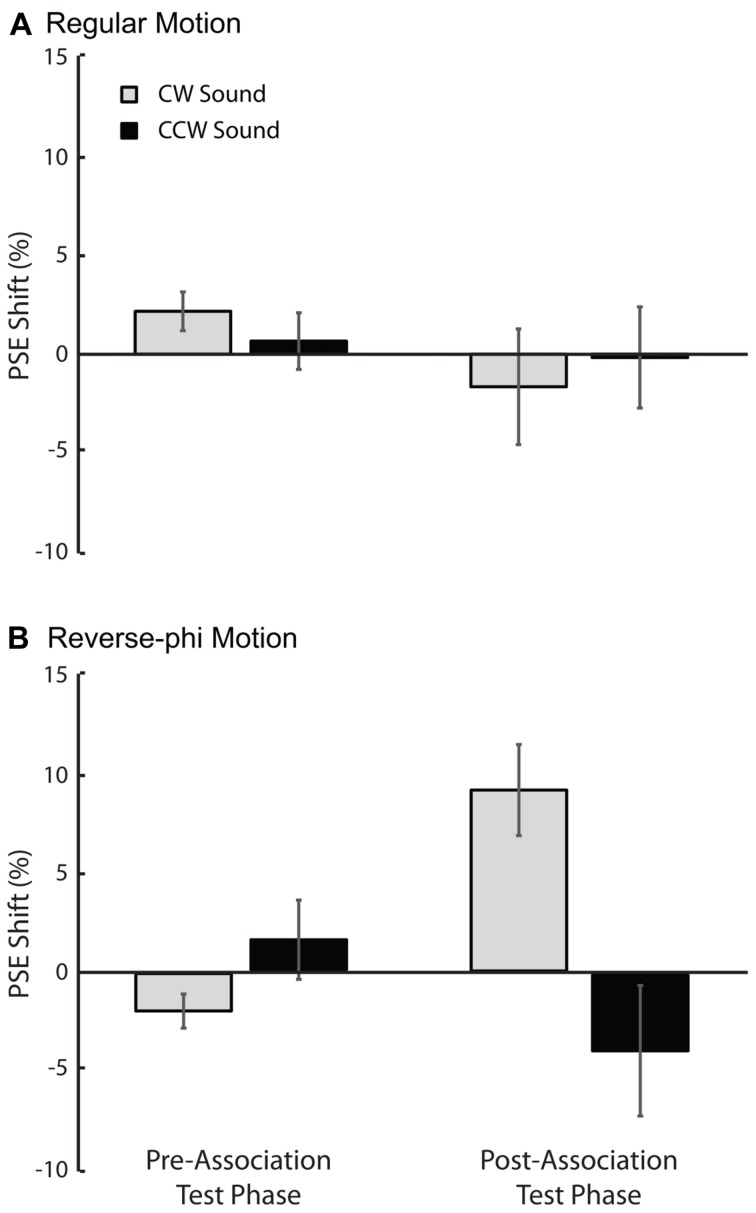
**Results of Experiment 2 (reverse-phi motion used in the association phase). The averaged PSE shifts of all observers as a function of test phase.** **(A)** Pre-association and post-association data for regular motion. **(B)** Pre-association and post-association data for reverse-phi motion. The gray and black bars represent CW and CCW sound conditions, respectively. Error bars indicate +SEM.

As shown in **Figures 4 and 5**, the audiovisual association had different effects on the subsequent perceived directions of regular and reverse-phi motions [three-way repeated-measures ANOVA on PSE shifts motion type, test phase and sound condition as factors, three-way interaction *F*(1,5) = 8.601, *p* < 0.05]. However, the main effects of motion type [*F*(1,5) = 0.122, *p* = 0.741], test phase [*F*(1,5) = 0.152, *p* = 0.713] and sound condition [*F*(1,5) = 4.491, *p* = 0.088] were not significant. After the association phase, static tones biased the perceived direction of the reverse-phi motion in favor of the paired presentations of perceived directions of reverse-phi motion and static tones (**Figure 4D**). As opposed to the pre-association test phase [*F*(1,5) = 1.824, *p* = 0.235], this led to a significant difference between two sound conditions in the post-association [**Figure 5B**, simple main effect of sound *F*(1,5) = 8.954, *p* < 0.05]. On the other hand, static tones did not cause any consistent change in the perceived direction of regular motion [simple main effect of sound: pre-association test *F*(1,5) = 1.794, *p* = 0.238; post-association test *F*(1, 5) = 0.336, *p* = 0.587]. As in Experiment 1, we did not find any significant difference between the bright and dark conditions of regular motion (Supplementary Table S2).

### Motion Direction Discrimination

**FIGURE 6:**
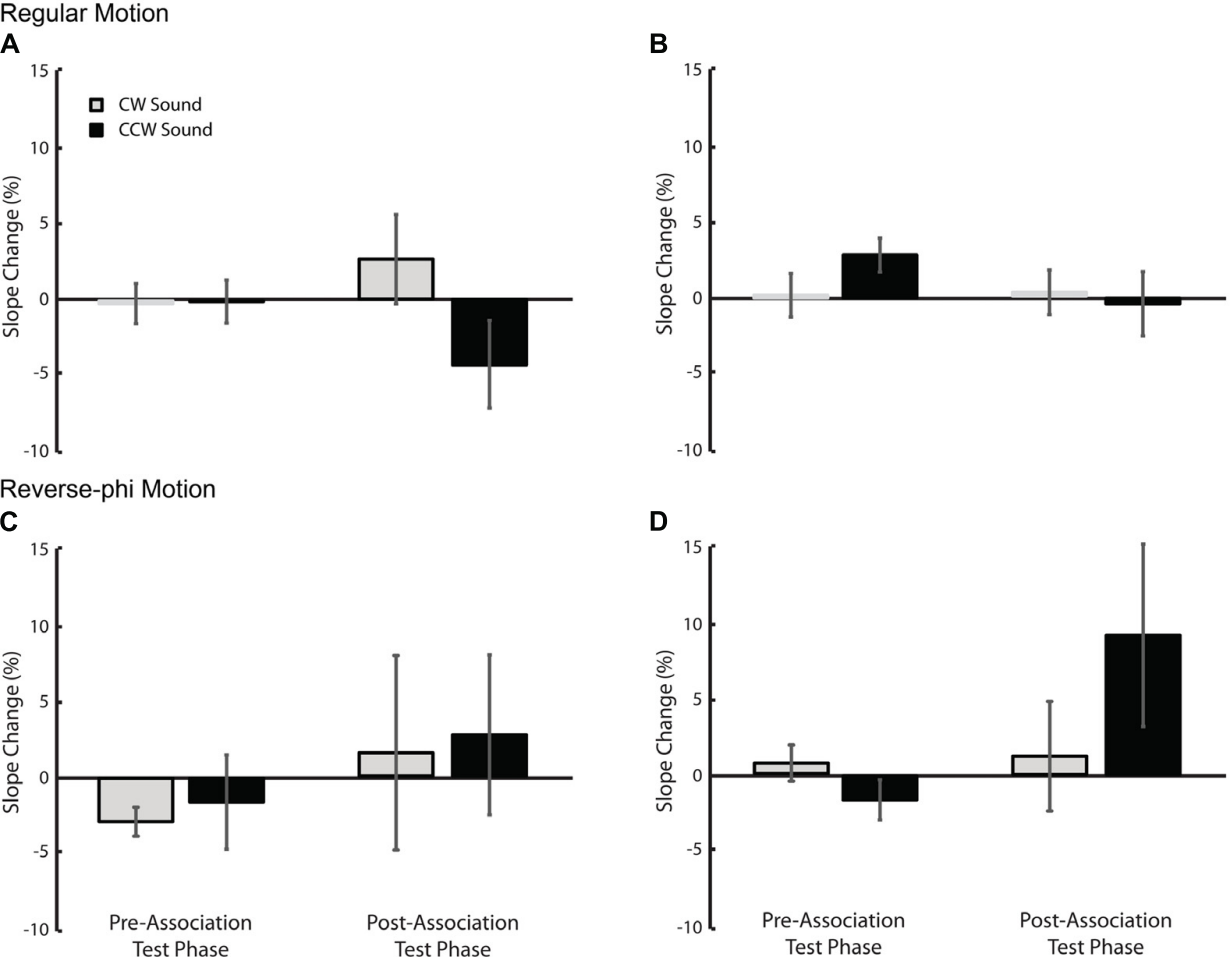
**The changes in slope values (in units of motion coherence level) relative to the visual-only condition.** Top panel: Experiment 1 **(A)** and Experiment 2 **(B)** regular motion data. Bottom panel: Experiment 1 **(C)** and Experiment 2 **(D)** reverse-phi motion data. Error bars indicate +SEM. Other conventions are the same as those in **Figures 3 and 5**.

The paired presentation of static tones with visual motion directed toward a given direction during association phase may also change subsequent direction discrimination thresholds. More specifically, in the post association test phase, static tones may increase the direction discrimination performance and decrease motion discrimination thresholds (i.e., steeper psychometric curves) for each sound condition relative to visual-only. We tested this possibility by using the changes in slope values (SDs) shown in **Figure 6**. A three-way repeated measures ANOVA (motion type, test phase and sound condition as factors) revealed no significant factor and interaction (Supplementary Table S3) for Experiment 1 (**Figure 6A,C**). For the second experiment, we found only a significant three-way interaction [Supplementary Table S4, *F*(1,5) = 40.102, *p* < 0.01]. Follow-up tests revealed only a simple main effect of sound [*F*(1,5) = 7.309, *p* < 0.05] in the post-association test of reverse-phi motion (**Figures 4D and 6D**). This suggests that the leftward shift of the psychometric function for the CCW sound condition (**Figure 4D**) is possibly confounded by the significant increase in the slope value. Overall, our results do not provide any consistent sound specific decrease in slope values and increase in direction discrimination performance due to audiovisual associations. If anything, the slope values tend to increase and direction discrimination performance tends to decrease after the association phase (for additional tests on raw slope values see Supplementary Tables S5 and S6).

## Discussion

Associations have been considered to play a role to disambiguate motion stimuli in noisy environments (Albright, 2012). Several studies have reported a compelling influence of audiovisual associations on ambiguous visual motion (Teramoto et al., 2010; Hidaka et al., 2011). After a brief association phase, static sounds can bias the perception of ambiguous visual motion and even induce a motion perception from static flickering objects. The mechanisms underlying these association induced effects have not been understood within the framework of motion processing hierarchy. In this study, we investigated whether audiovisual associations and their subsequent influences on motion perception were dependent on the involvement of higher-order attention-dependent position tracking mechanisms. We used regular and also reverse-phi motions to isolate low-level motion energy and to minimize the involvement of any explicit attentive tracking. After paired presentations of regular motion directions and static tones for around 8 min, static tones significantly biased the perceived directions of both regular and reverse-phi motions in favor of the exposed audiovisual pairing. In a follow-up experiment, we used reverse-phi motion during the association phase. We found that the association established between the (perceived) directions of reverse-phi motion and static tones affected only the subsequent perception of reverse-phi motion. We reasoned that if positional tracking were necessary for the association to be established and to impact the motion perception, then the perception of these low-level motion types should have been unaffected by static tones in the post-association test sessions as observed in the pre-association data. However, our results indicate significant changes in low-level motion perception (especially reverse-phi motion attributed to low-level motion energy mechanisms). It is also unlikely that the observed effects of audiovisual associations would simply be explained by response/decisional biases. If any decisional bias led to the observed changes in behavioral results, the association phase should have affected both motion types in a similar way. This is not the case when the reverse-phi motion is used for the association phase. Accordingly, the findings presented here suggest that audiovisual associations are not restricted to high-level attention based motion system, and that early-level motion processing has some potential role. In what follows, we discuss our findings with respect to their implications to visual motion processing, audiovisual (more generally multisensory) associations and interactions.

### Regular Versus Reverse-phi Motion Types

When we compare the outcomes from both experiments, it is obvious that the association phase with regular motion (Experiment 1: **Figure 3**) led to greater perceptual biases than the one with reverse-phi motion (Experiment 2: **Figure 5**). As mentioned above, the association phase with reverse-phi motion did not even bias the perceived direction of regular motion. Previous studies showed that adaptation to different types of visual motion can result in aftereffects with distinct characteristics. These distinctions have been mostly explained by differences between the mechanisms involved in processing visual motion types (Mather et al., 1998). Even though the tracking of individual dot positions was difficult for both regular and reverse-phi motion types in our experiments, it is still possible that regular phi motion might engage attention based position tracking more than the reverse-phi motion does. This minimal involvement of high-level attention based system may result in a more effective association phase for the regular phi motion. Another possibility is that there might be differences in the way these motion types drive the low-level motion detectors. More recently, low-level detection of regular and reverse-phi motions have been found to be different and it is suggested that these motion types drive low-level motion detectors in different ways (Bours et al., 2007). The differences found between low-level motion detection of regular and reverse-phi motion could also account for the differences observed in the audiovisual formed associations based on these two different motion types used in our study. Future studies are required to characterize the differences between the two audiovisual association types and to understand how they are linked to the motion processes involved.

### Low-Level and High-Level Processes in Visual Motion

Our findings demonstrate for the first time that the association established between visual motion directions and static tones impact the perceived direction of visual motion even when attentive tracking is ruled out. An interesting issue is whether these behavioral findings provide some information about which motion areas are involved in these audiovisual associations. Using functional imaging and motion stimuli which selectively tap into different motion systems, several studies identified distinct cortical areas involved in low-level pre-attentive and high-level attention based motion processing (Claeys et al., 2003; Ho and Giaschi, 2009a). Although lower visual areas (V1, V3A, and hMT+) appeared to be sensitive to all types of visual motion, several cortical areas were selectively activated by the motion types engaging low-level pre-attentive and high-level attentive tracking mechanisms prefentially (Claeys et al., 2003). Building from these findings, Claeys et al. (2003) identified the neuroanatomical substrates for these motion-processing systems in the human brain: the contralateral low-level motion energy system, extending from hMT+ into dorsal inferior parietal sulcus (IPS) and superior temporal sulcus (STS), and the bilateral higher level saliency-based system in the inferior parietal lobe (IPL). Moreover, IPS activation for the visual motion that increase the involvement of attentive tracking of salient visual features has been reported as significantly higher than for the one restricting any explicit attentive tracking (low-level visual motion), suggesting that this region represents a further stage than V3A and hMT+ in the motion processing hierarchy (Ho and Giaschi, 2009a). Based on these studies, our psychophysical results suggest that the influences of audiovisual associations on motion processing are not restricted to higher-order motion areas (e.g., IPL) mediating attention-based position tracking. They imply that lower visual motion areas (V1, V3A, and hMT+) are probably involved in audiovisual associations.

Our findings are in line with converging evidence showing that associative plasticity is not restricted to high-level visual motion processing and also present for neurons at early processing stages (Albright, 2012). By using a visual paired association task to link the direction of a static arrow with the direction of a random dot displays, Schlack and Albright (2007) found that many MT neurons exhibit selectivity for the direction of the static arrow. This property was not seen before the association between the static arrow and motion direction was formed. In addition to the relatively early-level plasticity in MT neurons, Fitzgerald et al. (2011) reported more profound changes in the selectivity of LIP neurons (Lateral Intraparietal cortex, part of monkey IPS) not only by associations between motion directions but also by other learned associations between pairs of arbitrarily chosen static shapes. More importantly, in a recent study, Zilber et al. (2014) trained human observers for 80 min to discriminate visual motion directions. They observed a great improvement in performance when the visual stimuli were presented with coherent acoustic textures, but not when presented with auditory noise. The MEG data collected in tandem revealed cortical plasticity in hMT+ reflecting the perceptual improvement associated with coherent auditory stimuli.

There have been numerous studies examining interactions between sound and visual motion (for a review see Soto-Faraco et al., 2003). Several illusions have demonstrated the strong influence of auditory stimuli on the perception of visual motion (e.g., Sekuler et al., 1997; Freeman and Driver, 2008; Hidaka et al., 2009). Of more direct relevance to our current study, many recent studies have suggested that the temporal and spatial information provided by audition is used at early stages of motion processing (Alink et al., 2008; Scheef et al., 2009; Kafaligonul and Stoner, 2010, 2012; Ogawa and Macaluso, 2013; von Saldern and Noppeney, 2013). That is to say, auditory information may interact with spatiotemporal processing of motion (low-level motion energy) at lower visual areas which have been traditionally thought to be unisensory (V1 and/or MT). The behavioral results presented here extend this recent view by showing that not only explicit stimulus properties of sound but also the audiovisual associations acquired through a brief exposure phase are also involved in shaping low-level visual motion perception, and they may be used at early stages of motion processing.

### Potential Neural Mechanisms

Our results do not preclude high-level processes (e.g., attention-dependent positional tracking), but support the view that low-level motion processing has a role in multisensory associations. The involvement of lower motion areas can take place in various forms. In general, associations are believed to be mediated by a strengthening of connectivity between the relevant neural representations (Albright, 2012). Neuroanatomical studies show some evidence of sparse connections between auditory and primary visual cortices (Falchier et al., 2002; Clavagnier et al., 2004; Cappe and Barone, 2005). It has been suggested that these connections may mediate early-level crossmodal interactions and auditory- or visual-driven phase resetting found in primary sensory cortices (Schroeder and Foxe, 2005; Kayser et al., 2008; Mercier et al., 2013). Accordingly, repeated exposure to static tones and moving stimuli in different directions may result in a strengthening of this early “crosstalk” between auditory and visual cortical areas (i.e., the lateral feedforward connections between auditory and lower visual motion areas). Vetter et al. (2014) have recently found that sounds can also induce mental imagery and hence, activate early visual cortex by feedback from non-retinal resources. In a similar way, static sounds may eventually elicit a pictorial recall of motion direction due to the paired presentations of static sounds with motion directions many times (the brief audiovisual association phase). Therefore, the influence of audiovisual associations on low-level visual motion may be mediated by a strengthening of top–down feedback connections from multisensory association and/or non-sensory areas to lower visual areas (Petro et al., 2014).

## Conclusion

In conclusion, the present study demonstrates that the association established between visual motion directions and static tones significantly affects low-level visual motion perception. These findings, in conjunction with a variety of related converging findings, suggest that audiovisual associations are not restricted to later stages of motion processing and lower visual motion areas has some potential role. Future neuroimaging and neurophysiological studies are needed to explicitly test whether audiovisual associations are restricted to higher-order visual areas and whether the audiovisual associations are mediated by feedforward lateral connections, top–down modulatory feedback or both.

## Conflict of Interest Statement

The authors declare that the research was conducted in the absence of any commercial or financial relationships that could be construed as a potential conflict of interest.

## Footnotes

1 Later studies called this paradigm as “sound contingent motion aftereffect” (Hidaka et al., 2011; Kobayashi et al., 2012a,b). In this study, we preferred to use the term “audiovisual associations” due to the following reasons: (i) aftereffects usually result in a bias opposite to the exposed direction and they are considered as a product of the recalibration of the sensory system, (ii) as pointed out in several studies (Seitz et al., 2007; Kim et al., 2009; Albright, 2012; Murphy et al., 2014), associations can be established implicitly (i.e., without any conditional reinforcement) at short time-scales.

2 An additional motion mechanism (second-order motion system), which is essential to extract the motion of features not defined by differences in luminance, is also supported by theoretical and experimental evidence (Lu and Sperling, 1995b).
