# Supplementary material for: Audiovisual associations alter the perception of low-level visual motion
Source: Front Integr Neurosci. 2015 Mar 31;9:26. doi: 10.3389/fnint.2015.00026 (PMC4379893; doi:10.3389/fnint.2015.00026)
Supplement: Supplementary file 1 [file table_1.pdf]

SUPPLEMENTARY MATERIAL

Table S1. Three-way ANOVA on regular motion PSE shifts in Experiment 1. The PSE shifts for bright and dark dot conditions were estimated separately.

**Factor/Interaction**	**Degrees of freedom**	**F**	**p**
Dot Polarity (bright/dark)	1,4	1.058	0.362
Test Phase (pre-/post-association)	1,4	0.353	0.584
Sound Condition	1,4	2.546	0.186
Dot Polarity × Test Phase	1,4	2.163	0.215
Dot Polarity × Sound Condition	1,4	5.712	0.075
Test Phase × Sound Condition	1,4	6.908	0.058
Dot Polarity × Test Phase × Sound Condition	1,4	2.873	0.165

Table S2. Three-way ANOVA on regular motion PSE shifts in Experiment 2. The PSE shifts for bright and dark dot conditions were estimated separately.

Factor/Interaction	**Degrees of freedom**	**F**	**p**
Dot Polarity (bright/dark)	1,5	2.104	0.207
Test Phase (pre-/post-association)	1,5	2.046	0.212
Sound Condition	1,5	0.042	0.845
Dot Polarity × Test Phase	1,5	0.435	0.539
Dot Polarity × Sound Condition	1,5	2.836	0.153
Test Phase × Sound Condition	1,5	0.773	0.419
Dot Polarity × Test Phase × Sound Condition	1,5	0.504	0.51

Table S3. Three-way ANOVA on slope changes in Experiment 1.

**Factor/Interaction**	**Degrees of freedom**	**F**	**p**
Motion Type	1,4	0.083	0.788
Test Phase (pre-/post-association)	1,4	0.499	0.519
Sound Condition	1,4	1.438	0.297
Motion Type × Test Phase	1,4	0.354	0.584
Motion Type × Sound Condition	1,4	3.428	0.138
Test Phase × Sound Condition	1,4	0.71	0.447
Motion Type × Test Phase × Sound Condition	1,4	1.626	0.271

Table S4. Three-way ANOVA on slope changes in Experiment 2.

Factor/Interaction	Degrees of freedom	F	p
Motion Type	1,5	0.378	0.565
Test Phase (pre-/post-association)	1,5	0.757	0.424
Sound Condition	1,5	5.241	0.071
Motion Type × Test Phase	1,5	2.089	0.208
Motion Type × Sound Condition	1,5	0.488	0.516
Test Phase × Sound Condition	1,5	4.162	0.097
Motion Type × Test Phase × Sound Condition	1,5	40.102	*0.001*

Table S5. Three-way ANOVA on raw slope values in Experiment 1. After the association phase, there was slight increase in slope values for both regular (pre-association: 17.45 $\pm$ 2.23; post-association: 18.88 $\pm$ 2.63) and reverse-phi motion (pre-association: 16.13 $\pm$ 4.13; post-association: 22.60 $\pm$ 4.70). Even though there was no significant interaction between factors, we also performed two-way ANOVAs on regular and reverse-phi motion separately. They revealed that the test phase was significant for reverse-phi motion ($F(1, 4) = 16.852$, $p = 0.015$) but not for regular motion ($F(1, 4) = 5.344$, $p = 0.082$). This suggests that the significant effect of test phase observed in three-way ANOVA was mostly due to the reverse-phi motion data. Follow-up tests for simple main effects and post-hoc tests did not reveal any significant sound specific changes.

Factor/Interaction	Degrees of freedom	F	p
Motion Type	1,4	0.054	0.828
Test Phase (pre-/post-association)	1,4	14.871	*0.018*
Sound Condition (2 sound + visual-only)	2,8	0.581	0.612
Motion Type × Test Phase	1,4	2.05	0.225
Motion Type × Sound Condition	2,8	1.313	0.389
Test Phase × Sound Condition	2,8	0.712	0.558
Motion Type × Test Phase × Sound Condition	2,8	2.22	0.256

Table S6. Three-way ANOVA on raw slope values in Experiment 2. As in Experiment 1, there was an increase in slope values for both regular (pre-association: 14.59 + 0.92; post-association: 16.21 + 0.84) and reverse-phi motion (pre-association: 17.50 + 2.57; post-association: 31.20 + 8.10). However, the test phase was not significant. An additional two-way ANOVA on reverse-phi slope values did not reveal a significant effect of test phase ($F(1, 5) = 4.485$, $p = 0.088$). To disentangle the source of three-way interaction below, we conducted additional two-way ANOVAs, tests for simple main effects and post-hoc tests. In brief, we only found a significant simple main effect of sound at the post-association phase of reverse-phi motion ($F(2, 10) = 7.269$, $p = 0.047$) and a follow-up post-hoc test revealed significant difference between CW and CCW sound conditions ($p < 0.05$). The slope values for CCW sound condition were bigger than the ones for CW sound condition.

**Factor/Interaction**	**Degrees of freedom**	**F**	**p**
Motion Type	1,5	2.861	0.152
Test Phase (pre-/post-association)	1,5	5.517	0.066
Sound Condition (2 sound + visual-only)	2,10	2.138	0.234
Motion Type × Test Phase	1,5	3.338	0.127
Motion Type × Sound Condition	2,10	0.202	0.825
Test Phase × Sound Condition	2,10	2.068	0.242
Motion Type × Test Phase × Sound Condition	2,10	35.873	*0.003*
